# A Perspective on Age Restrictions and Other Harm Reduction Approaches Targeting Youth Online Gambling, Considering Convergences of Gambling and Videogaming

**DOI:** 10.3389/fpsyt.2020.601712

**Published:** 2021-01-25

**Authors:** Jing Shi, Michelle Colder Carras, Marc N. Potenza, Nigel E. Turner

**Affiliations:** ^1^Institute for Mental Health Policy Research, Center for Addiction and Mental Health, Toronto, ON, Canada; ^2^School of Rehabilitation Science, McMaster University, Hamilton, ON, Canada; ^3^JHU Global mHealth Initiative, Department of International Health, Johns Hopkins Bloomberg School of Public Health, Baltimore, MD, United States; ^4^Department of Psychiatry and Child Study Center, Yale University School of Medicine, New Haven, CT, United States; ^5^Connecticut Council on Problem Gambling, Wethersfield, CT, United States; ^6^Connecticut Mental Health Center, New Haven, CT, United States; ^7^Department of Neuroscience, Yale University, New Haven, CT, United States; ^8^Dalla Lana School of Public Health, University of Toronto, Toronto, ON, Canada; ^9^Campbell Family Mental Health Research Institute, Center for Addiction and Mental Health, Toronto, ON, Canada

**Keywords:** gambling, harm reduction, gaming, video games, addictive behavior, child, adolescent, Internet

## Abstract

Internet gambling has become a popular activity among some youth. Vulnerable youth may be particularly at risk due to limited harm reduction and enforcement measures. This article explores age restrictions and other harm reduction measures relating to youth and young adult online gambling. A systematic rapid review was conducted by searching eight databases. Additional articles on online gambling (e.g., from references) were later included. To place this perspective into context, articles on adult gambling, land-based gambling, and substance use and other problematic behaviors were also considered. Several studies show promising findings for legally restricting youth from gambling in that such restrictions may reduce the amount of youth gambling and gambling-related harms. However, simply labeling an activity as “age-restricted” may not deter youth from gambling; in some instances, it may generate increased appeal for gambling. Therefore, advertising and warning labels should be examined in conjunction with age restrictions. Recommendations for age enforcement strategies, advertising, education, and warning labels are made to help multiple stakeholders including policymakers and public health officials internationally. Age restrictions in online gambling should consider multiple populations including youth and young adults. Prevention and harm reduction in gambling should examine how age-restriction strategies may affect problem gambling and how they may be best enforced across gambling platforms. More research is needed to protect youth with respect to online gambling.

## Introduction

People with gambling problems typically meet criteria for hazardous gambling, betting or gambling disorder in the International Classification of Diseases, 11th edition (1, 2). Global estimates of 10- to 24-year-olds suggest 0.2–12% of youth and young adults experience gambling problems (3, 4), with an additional 8–14% at risk for developing gambling problems (5). Online gambling prevalence in 13- to 24-year-olds range between 4 and 24% (6). It is estimated that 2.5% of youth, or 18.1% of those who gambled online, experiences problematic gambling (7).

Gambling-related harms may be experienced by those who gamble, associated individuals, and communities through social systems and/or health systems costs (8, 9). The Conceptual Framework of Harmful Gambling proposed that the definition of harmful gambling is, “any type of repetitive gambling that a person engages in that leads to (or aggravates) recurring negative consequences, such as significant financial problems, addiction, or physical and mental health issues.” p. 4 (9). Such harms may include financial and interpersonal problems (10, 11), nongambling psychiatric disorders (12, 13), and they could increase strain on welfare systems and generate economic harms in the community (8). Youth and young adults may be particularly susceptible to problem-gambling-related harms, especially to online gambling since it is often fast-paced and easily accessible (6, 14, 15). As new forms of online gambling emerge, the issue of problem gambling in youth may become more prevalent and differ significantly from land-based gambling (e.g., at in-person venues like casinos).

Enforcement of harm reduction measures related to online gambling varies. Youth may gamble online by clicking to indicate they are “over 18 years old.” Furthermore, a convergence of gambling and videogaming has implications for youth gambling. Limited or no age restrictions for online games such as free-to-play slot machines may allow youth early opportunities to engage in gambling-like activities that may lead to gambling problems (16). Social casino games (SCGs) that involve virtual currency may lead to monetary gambling (17, 18). Other videogaming-related features such as loot boxes^1^ (19) and skins betting^2^ (20) offer non-monetary rewards with in-game value that may also have monetary value. A convergence between gambling and videogaming platforms may facilitate behavioral involvement across networks and consoles, providing robust access to gambling-like activities (21). It is therefore important to understand how best to use age restrictions and other harm-reduction measures for online gambling and videogaming in preventing or minimizing online-gambling-related harms.

## Methods

A rapid review was conducted for age restrictions and warning labels in youth gambling by searching Cochrane, PsychInfo, Embase, Medline, Child Development and Adolescent Studies, PAIS, Web of Science, and Social Care Online between February 2–18, 2020. In order to put this perspective narrative into context, additional articles on online gambling were included between March to November 2020 through database and internet searches. Articles on adult gambling, land-based gambling, and other potentially risky behaviors were considered. Here, “youth” refers to people under the legal gambling age; however, young adults are also considered since some youth studies included people up to age 25. Further rationale for including young adults is described below.

## Youth and Young Adult Online Gambling

Youth are often exposed to gambling at early ages, and many gamble online (22). The idea that gambling is potentially harmful for youth is longstanding. In 1978, Cornish (23) stated that it is dangerous to introduce gambling to youth because their lives are not yet structured by the constraints, obligations, and rewards that adults have which act to prevent excessive involvement with gambling. An early age of gambling onset is associated with developing gambling problems, particularly for males (24–26), and more severe gambling problems later in life (27). Early gambling also is associated with serious negative psychological, social, financial, and substance use problems (28–30).

Adolescents are more inclined to participate in, and underestimate the risk of risk-taking behaviors such as substance use and online gambling (3, 31). Failure to address youth concerns may lead to negative impacts (15, 32). However, young adults (ages 18–24) may also be at elevated risk given neurodevelopmental processes underlying risk-taking and addictive behaviors. Emotional regulation, logic and other processes are not fully developed by young adulthood (33). Therefore, poor decision-making may lead young adults to take more risks and act more impulsively when gambling (33). For example, individuals aged 18–20 years are particularly likely to chase losses and bet more than they can afford (33). This may present a problem because young adults up to 25 years old may be overlooked by gambling legislation in several countries that have legal age restrictions for those under 18- to 21-year-olds.

Youth and young adult online gambling is a growing concern as studies suggest that this demographic is shifting away from land-based gambling to online gambling (34–36). Youth are also moving from social gambling with friends to solo gambling online that is available across time and locations (14). This is particularly concerning since, for youth, online gambling has been associated more with problem gambling than land-based gambling. International studies found higher proportions of problem gambling among youth who gambling online vs. non-online (34, 36–38). Jurisdictions should enact and enforce strict measures to stop early gambling in order to prevent the onset of gambling problems later in life.

## Convergence of Gambling and Videogaming

Videogames that include gambling-like features or free-to-play gambling-related games, like SCGs, vary with respect to age restrictions and their enforcement (39–41). Gambling-related games without monetary wagering typically do not fulfill legal criteria for gambling (39, 40). Access to land-based or online video, amusement, and slot machines may have ambiguous age restrictions, and children under 16 years old sometimes have legal access (41). A Canadian sample of youth in grades 9–12 found 12.4% had played SCGs in the past 3 months. These youth were more prevalently classified as experiencing problem gambling (18). A similar study in the United States found that ~10% of adolescent gamblers reported gambling at a casino, with estimates of 40% among those with gambling problems (42). In a Hong Kong school-based survey, 71.4% of individuals who gambled online reported earlier participation in games on free-to-play websites. These individuals were likely to view gambling as safe and healthy entertainment (36). However, free-to-play gambling-related games have been linked to gambling for money and problem gambling in youth (14, 16, 22, 43). Furthermore, microtransactions in simulated gambling-related games have been associated with subsequent gambling (35, 42). However, more longitudinal research is needed.

Forms of gambling may be incorporated into videogames and vice versa, blurring boundaries (20, 43). For example, some governmental and regulatory bodies consider loot boxes as gambling elements in videogames (44). Individuals who play videogames problematically have reported using online videogames and digital platforms to gamble (45). For example, some in-game items (even non-game-enhancing, cosmetic ones) may be exchanged for significant real-world money (46). Loot boxes, skins, and other random-chance features are considered to have similarities to gambling. These are found in games deemed suitable for youth as young as 8-years-old (47). Among the top 100 grossing videogames, loot boxes were prevalent, especially on mobile platforms, with these videogames often available to children 12 years or older (48).

Videogaming features such as loot boxes (19, 48–51) and skins betting (20) may be gateways to gambling and gambling problems in youth. Youth participating in skins betting and gambling may be at elevated risk for gambling-related harms (20, 48). A qualitative analysis of 16- to 18-year-olds who purchased loot boxes suggested that reasons for purchases were similar to reasons for engaging in gambling (51). These included wanting to advance in videogames more quickly, raising money, excitement, and escaping from stress (19, 51). Such findings indicate that age restrictions and harm-reduction measures should be considered for videogames that contain gambling/gambling-like elements. Healthcare professionals should understand the natures of videogames played in relation to their clients'/patients' lives (44). Contexualizing youth videogaming and gambling may be critical in preventing online gambling problems (14).

The role of virtual communities for gambling and videogaming should be considered during prevention and treatment of gambling/videogaming problems, especially for women (52). Identification within virtual communities may considerably influence in-game spending behaviors (52). Additional input is needed from game developers and rating boards (50). Online videogaming and gambling providers could take proactive roles in identifiying and excluding gambling youth. Similar approaches may be applicable to identifying, intervening and limiting at-risk gambling/videogaming (31). Providers could also include links to online counseling, peer-support chats, educational materials, and virtual communities that may serve as protection against excessive use (31, 52). Policymakers could consider placing limits on chance-items and use other controls that are traditionally used in gambling settings to limit youth spending and prevent youth engagement (49, 50, 53, 54). Additional harm-reduction measures are discussed below.

### Effectiveness of Age Restrictions as a Harm-Reduction Measure

Limited research exists on the effectiveness of age restrictions on youth gambling, despite theoretical support (55). While age restrictions may prevent problem gambling or related harms (56–58), their effectiveness have largely been untested. Effectiveness of legal age limits appears largely inferred based on worldwide implementation (58). However, a global solution may be unfeasible. Customers typically prefer easy access, gambling and videogaming corporations are often profit-driven, and many governments take some revenue either directly or indirectly through taxation from gambling (58). Therefore, harm reduction or prevention of problem gambling by limiting the number of customers and the profits from these customers may not be the first solution considered.

Effectiveness of age restrictions on gambling may be influenced by public awareness and enforcement. A Finnish study found that teacher awareness for the minimum legal age of gambling was not as accurate as for purchasing alcohol, purchasing cigarettes, or driving a car (59). Similarly, in Canada, youth gambling was viewed as requiring less attention than other risk behaviors by teachers (60) and parents (61). With social acceptance of gambling, few caregivers may be aware of potential risks of early gambling onset (61). Underaged youth often participate in illegal gambling despite age restrictions (36, 41, 62, 63). Infringements against or disregard for age restrictions appear more common among males (64).

As with tobacco and alcohol, age restrictions are only effective when rigorously enforced (55, 65). There currently appears to be inadequate enforcement of age restriction regulations across multiple gambling activities (24, 41, 66). Enforcing age restrictions for online gambling may be particularly difficult. Underaged individuals who gamble may be committing credit card fraud or are being supported by older friends and relatives to gamble online (31, 36). There are currently few safeguards to protect underaged individuals from gambling, and there have been calls for strict verification systems to be implemented (15, 36). Strategies used to enforce age restriction for in-person gambling may work for online gambling, although challenges exist in applicability (Table 1).

**Table 1:** Age restrictions enforcement strategies.

**Recommendation**	**Description**
Use Age Verification	Compliance with age limits is poor (65). Age verification with personal identifications systems, by having users log on using a national identification number, can prevent underage gambling (14). Verifying age verbally *and* requesting identification in-person for land-based venues appear important. Simply asking the age of an individual is largely ineffective in enforcing underaged gambling. Compliance rates were the following for asking for: age only (0%); identification only (67%); age and identification (75%) (58).
Use Fines	Introduce fines for non-compliance may increase effectiveness. In the Netherlands, underaged individuals who gamble may be fined (19). Fines may also be introduced to vendors of gambling products.
Restrict visibility	Relaxation of gambling controls in the U.K. allowed retail outlets (e.g. newsagents, convenience stores, petrol stations, etc.) to have online terminals to sell lottery tickets including instant (scratch) lottery tickets. This gives vulnerable populations exposure and increased opportunity to participate in gambling (41).
Restrict convenient access	More access to gambling was noted in off-site locations such as gambling stores like the ones mentioned above (0% compliance rate) compared to on-site locations such as casinos (14% compliance rate) (58). Access to public gambling machines presents a potential threat for gambling disorder in minors as entry into casinos is limited to individuals 18 years or older in many jurisdictions (67).
Restrict availability	In theory, legal age limits should act to limit availability of gambling products. Enforcement of laws has been easier when limiting availability of slot machines within dedicated gambling areas (24, 68). However, setting limits may potentially increase gambling problems for some people; stakeholders should examine directly the consequences of placing limits if and when they do (69).
Use warning labels and messages	Warning labels are effective at modifying gambling behavior (70). Messages are informative to consumers, and if applied appropriately, they have the potential to reduce harm (70). In a laboratory setting with undergraduate students, those who received warning messages on common irrational gambling beliefs demonstrated significantly fewer irrational beliefs and less risky gambling behavior than those in the control condition who received messages on the history of roulette (71).

### Raising Age Minimums

Research examining effectiveness of raising legal ages for gambling is limited; however, a review suggests that raising minimal ages may reduce gambling-related harms (72). Finnish studies examined effects of raising the legal minimum age to gamble from 15 to 18 years with an interest in protecting youth from gambling-related harms (55, 68, 73, 74). Unsurprisingly, 18-year-olds who were not targeted by the age increase showed no significant changes in gambling activity (74). The intervention was successful in reducing lottery and slot-machine gambling for the 15- to 17-year-old age group and, interestingly, also the 18- to 19-year-old age group 3 years post-legislation (73). Nonetheless, underaged gambling was still occurring in about 13% of youth (55). Online gambling for all age groups, except for underaged 15- to 17-year-olds, increased. Online gambling was rare in the 15–17 age group [4%] (68), perhaps related to difficulties in obtaining credit cards to gamble.

In sum, the Lotteries Act enacted in Finland on October 1, 2010 that raised the minimum age limit for gambling from 15 to 18 years of age helped decrease adolescent gambling and problem gambling between 2011 and 2015 (59). Teens who were still gambling experienced significantly less gambling-related harms 6 years after raising the age minimum (73). Therefore, negative consequences experienced by youth from gambling may be less prevalent after raising the age minimum (74). Follow-up is required to examine longer-term effects, especially on online gambling.

### Warning Labels

Warning labels and advertising may reduce youth online gambling (75). However, few studies have examined intervention effectiveness in real-world gambling settings (76). Consumers do not appear “desensitized” to multiple warning messages (77, 78). Increased exposure to warnings may be beneficial in preventing youth online gambling. Also, providing only knowledge about gambling on warning labels does not necessarily impact gambling behavior. When gambling odds were on warning messages to alter irrational beliefs about winning, gambling behavior did not change significantly (79). A study with students (ages 14–17 years) found age-related warning labels with highly caffeinated food and drinks were similarly ineffective (80). In some cases, warning labels increased appeal of products (56% for videogames) (81). Gambling products were not part of this study, and therefore, it is uncertain whether such warnings on gambling products would increase gambling appeal to youth. Warning-label features that may be applicable to online youth gambling are discussed in Table 2.

**Table 2:** Summary of recommendations for warning labels.

**Recommendation**	**Description**
Feature a trustworthy source	Although a U.S. study on youth and cigar warnings found no differences between sources of warning labels (82), a gambling study found that a trustworthy source for the warning label is important for its believability. Medical sources were found to be more effective than governmental sources (83). Moreover, a source related to the gambling provider had almost the same effect as no source (83).
Place warnings on each gambling machine, table, scratch ticket, and gambling website	An online survey at a U.S. college on waterpipe use showed that the location of the placement of the warning was important (84). In relation to gambling, this may mean that harm-based messages should be placed in noticeable locations where potential consumers can see them easily and frequently. For online gambling, placement on the website and how warnings are incorporated into experiences on the website are likely important considerations. Making labels conspicuous rather than discrete appears important (85).
Use pop-up style messages rather than static messages	Pop-up messages may have significantly more impact on thoughts and behaviors than static messages (86, 87). In one study, pop-up messages were recalled more immediately after gambling sessions and at a 2-week follow-up (86). Pop-up-style messages may be optimal when displayed in the center of screens, when they interrupt gambling, and when they require participant action to remove them (70).
Use honest warnings regarding negative consequences	Greater understanding of negative consequences may create more fear in people who gamble, which may then prompt them (at least in the short term) to consider risks that they are facing (83). However, long-term effects are less well known. For people with gambling problems, adults who had lower experiential avoidance were more responsive to fear-inducing warnings than were those with higher experiential avoidance (88).
Use simple descriptive messages rather than longer and more complex warnings	Longer patient-information warnings about gambling behaviors may be overwhelming (70) and, therefore, ineffective (89).
Use messages that discuss money spent	Messages that discuss money spent may have the greatest impact on gambling behavior (90).
Create tailored labels/messages	In a U.S. anti-substance-use study, youth were asked to design their own messages. The more time that youth had, the more persuasive their messages were in deterring youth substance use (91). In a focus group study with First Nations and Metis youth, messages tailored to cultural backgrounds and gender were found to be more effective (92). In a gambling study with young adults, people who gambled responded better to messages about their own gambling and expertise, with people engaging in “skill-based” gambling responding to messages on odds of winning and outcomes over time (93). Tailored message could also encourage self-appraisal rather than provide informative messages. Although both messages that encouraged self-appraisal and messages that were informative reduced gambling through behavior change (90), messages that ask people who gamble to self-appraise had significantly greater impact on thoughts and behaviors (86).
Use pictorial rather than text warning labels	Graphic warning labels (GWLs) were more effective than text-only warnings or personal testimonials (76, 94–96). Youth, especially those of younger age, tended to pay more attention to images than to text (97, 98). Images that created greater reactance or negative emotions (85, 99–102), were in full color (103), and used larger warnings with pictures (104) were found often to be more effective. Other studies found only comparable levels of negative emotions elicited by GWLs (103) and that they were generally more effective for those who already gambled. Similar studies were supported in the smoking literature where the effects of GWLs were lower for non-smoking than smoking individuals (95, 102). GWLs may not be an effective deterrent for youth who are not yet gambling. More research is needed to determine appropriate GWLs for youth videogaming and gambling.
Present two-sided messages	Framing warning messages as a “loss” or in a negative way, rather than what can be “gained” by not participating in the risky behavior, may be effective as a prevention method for adolescents (105). However, this may be different in the nutrition industry. Across three studies, dieting individuals who saw a negative message on unhealthy foods had an increased desire for consumption of those foods. Non-dieting individuals ignored the messages. In some cases, two-sided messages rather than just a negative message, may be a better option (106). An example used by the food industry is, “All dessert tastes good, but is bad for your health” p. 175 (106). Gambling products were not a part of these studies; however, framing two-sided messages may be a cautious way to proceed. A two-side message for gambling may be, “You can win money, but you can also lose *everything*.”

### Advertising and Education

Advertising and promotion of educational interventions warrant further study (14, 15). Interventions targeting youth gambling may fail without public awareness. When Finland raised age restrictions on gambling, mass media campaigns increased awareness and supported changes (55). Campaigns may use gambling websites, radio, physical posters in public spaces, online news, and social media platforms (31, 55). Conscientious marketing may help prevent under-aged involvement in online poker (16), especially when visibility of gambling advertisements contributes to people experiencing increased gambling accessibility (14). A UK study found harm-reduction messages were less visible than advertising (107). Recommendations for gambling advertising include:

1. Restricting advertising of online gambling (68, 108);
2. Including warning messages on all advertising and promotional materials (36);
3. Prohibiting marketing that targets underaged or vulnerable populations (73). This last point involves not depicting youth or people who look underaged participating in gambling activities (109, 110) and not implicitly or explicitly directing advertising at them (110). Increased education regarding risks should also be included in a comprehensive policy approach and harm-reduction guidelines (111).

While it may be nearly impossible to regulate all forms of online gambling, harm reduction in the form of educational awareness may help. Mass media campaigns and educational material that can inform youth of negative health effects could be implemented (31, 75, 108). Education to promote awareness of gambling risks could be implemented in schools and colleges, and incorporated into school curricula to prevent youth gambling and future gambling problems (31, 72, 112). Informational websites with links to treatment services and warnings to family/friends against providing funds to support youth gambling should also be considered (14, 36).

## Limitations

This perspective paper provides a narrative overview of literature related to online youth and young adult gambling and age restrictions. Online gambling may change as videogaming and gambling converge and new technologies are developed (113). Although this paper began as rapid review on age restrictions and warning labels for youth, additional literature was cited to contextualize youth online gambling. This paper should not be considered a comprehensive critical description of the entire literature.

## Conclusions

From the reviewed studies, there appears to be widespread adoption of legal age restrictions on gambling; however, studies of effectiveness pertaining specifically to online gambling appear limited. This may reflect indirect effects of harm-reduction regulations that primarily aim to denormalize and prevent youth from learning of financial and social rewards through gambling (114, 115). Enforcement of age restrictions, however, is another challenge. Future work surrounding prevention and harm reduction in online gambling should longitudinally examine optimal age restrictions and how they may be best enforced across the internet, considering adolescent/youth development. Current age restrictions should be consistently enforced to understand better their effects. In addition, further research is needed to reduce harms related to youth online gambling and gambling-related features in videogames. Early adoption of harm reduction measures including higher age restrictions for online gambling and for videogames with gambling-related features may be beneficial.

Evidence from research in gambling and related fields suggests that warning labels that simply state “age restricted” may not deter youth or may even increase appeal. Effective warning labels should consider tailored, strong, and colorful graphics that depict negative consequences of gambling. Messages that are simple and concise from a reliable source such as a medical organization may be effective with some youth. Balanced messages that tell two sides of the story (both positive and negative aspects of online gambling), are honest about negative consequences, discuss money spent, or encourage-self appraisal may also deter youth online gambling. Finally, youth may not become desensitized to warning labels and may require reminders as refreshments. Placing pop-up warning labels in noticeable areas where youth and other vulnerable populations may gamble online could be effective. However, direct examination of the effectiveness of each of these approaches for youth online gambling is needed.

## Data Availability Statement

The original contributions presented in the study are included in the article/supplementary material, further inquiries can be directed to the corresponding author/s.

## Author Contributions

JS wrote the first draft of the paper and worked with the other co-authors on subsequent drafts. All authors contributed to the editorial process and have approved the final submitted version of the manuscript.

## Conflict of Interest

MC serves on the advisory board of a veteran-serving organization focusing on video games and mental health. She is the CEO and founder of Gaming and Wellness Association, Inc., a non-profit dedicated to research and education about healthy video game play. MP has consulted for and advised Game Day Data, the Addiction Policy Forum, AXA, Idorsia, and Opiant/Lakelight Therapeutics; received research support from the Veteran's Administration, Mohegan Sun Casino, and the National Center for Responsible Gaming (no the International Center for Responsible Gambling); participated in surveys, mailings, or telephone consultations related to drug addiction, impulse-control disorders or other health topics; consulted for law offices and the federal public defender's office in issues related to impulse-control and addictive disorders; provided clinical care in the Connecticut Department of Mental Health and Addiction Services Problem Gambling Services Program; performed grant reviews for the National Institutes of Health and other agencies; edited journals and journal sections; given academic lectures in grand rounds, CME events and other clinical/scientific venues; and generated books or chapters for publishers of mental health texts. The other authors report no disclosures. The views presented in this manuscript represent those of the authors. NT has received funding from the Ontario Ministry of Health and Long Term Care, and Gambling Research Exchange. He has also acted as a consultant on gambling problems for various government and legal entities. He has received grant funding from the Ontario Lottery and Gaming (the crown corporation that manages gambling in Ontario) to evaluate one of their prevention initiatives, but otherwise has not received funding from the gambling industry. The contract included guarantees of independence and intellectual property rights for the researcher. The remaining author declares that the research was conducted in the absence of any commercial or financial relationships that could be construed as a potential conflict of interest.

## Footnotes

^1^Loot boxes are videogame features (often in the shape of a box) available in many game genres that one can find or purchase. They often contain a seemingly random mix of items, ranging from common to rare items. The rarer the item, the more valuable it typically is in the game. In some cases, a loot box may be found in-game but requires a key to open it—this key may be purchased or earned. A distinction from gambling is that loot boxes can create monetary losses but typically no monetary gains.

^2^Skins in videogames change the appearance of an item or character. For example, a skin may give your gun camouflage coloring, or give it the appearance of flames. Skins can be obtained through loot boxes, earned during gameplay, purchased with virtual currency and/or purchased with real money. For some videogames, skins become a valuable commodity that can be sold or used to place bets with on third-party websites.
